# Changes in the Personal Dignity of Nursing Home Residents: A Longitudinal Qualitative Interview Study

**DOI:** 10.1371/journal.pone.0073822

**Published:** 2013-09-12

**Authors:** Mariska G. Oosterveld-Vlug, H. Roeline W. Pasman, Isis E. van Gennip, Dick L. Willems, Bregje D. Onwuteaka-Philipsen

**Affiliations:** 1 Department of Public and Occupational Health, EMGO Institute for Health and Care Research, Expertise Center for Palliative Care, VU University Medical Center, Amsterdam, The Netherlands; 2 Department of General Practice, Section of Medical Ethics, Academic Medical Center, Amsterdam, The Netherlands; University of Nebraska Medical Center, United States of America

## Abstract

**Background:**

Most nursing home residents spend the remainder of their life, until death, within a nursing home. As preserving dignity is an important aim of the care given here, insight into the way residents experience their dignity throughout their entire admission period is valuable.

**Aim:**

To investigate if and how nursing home residents’ personal dignity changes over the course of time, and what contributes to this.

**Design:**

A longitudinal qualitative study.

**Methods:**

Multiple in-depth interviews, with an interval of six months, were carried out with 22 purposively sampled nursing home residents of the general medical wards of four nursing homes in The Netherlands. Transcripts were analyzed following the principles of thematic analysis.

**Results:**

From admission onwards, some residents experienced an improved sense of dignity, while others experienced a downward trend, a fluctuating one or no change at all. Two mechanisms were especially important for a nursing home resident to maintain or regain personal dignity: the feeling that one is in control of his life and the feeling that one is regarded as a worthwhile person. The acquirement of both feelings could be supported by 1) finding a way to cope with one’s situation; 2) getting acquainted with the new living structures in the nursing home and therefore feeling more at ease; 3) physical improvement (with or without an electric wheelchair); 4) being socially involved with nursing home staff, other residents and relatives; and 5) being amongst disabled others and therefore less prone to exposures of disrespect from the outer world.

**Conclusion:**

Although the direction in which a resident’s personal dignity develops is also dependent on one’s character and coping capacities, nursing home staff can contribute to dignity by creating optimal conditions to help a nursing home resident recover feelings of control and of being regarded as a worthwhile person.

## Introduction

Being admitted to a nursing home can have a large impact on peoples’ lives. This impact can be particularly profound when the transition from living more or less independently at home to living in a nursing home and being dependent is sudden [1,2]. These changes in body and living situation make nursing home residents vulnerable with regard to loss of personal dignity [3,4], a type of dignity that is subjectively experienced by an individual [5]. A German study discovered that nursing home residents placed their personal dignity under the constraints of the need for help and care into question, which could undermine their dignity if a resident was not able to obtain a new perspective on this phase of life [6]. A task for professionals is therefore to help residents in finding new strategies in their new living situation and to reinforce their coping skills [7].

The literature describes different coping strategies: problem-focused coping, in which one tries to do something about the problem itself [8] and emotion-focused coping, in which one tries to manage feelings that have arisen as consequences of a threat [9]. The latter is used when the outcome is judged to be unchangeable, as is often the case for frail old people living in nursing homes. Earlier studies found that nursing home residents attempted to preserve their dignity by various coping strategies, e.g. adjusting to and accepting the situation, focusing on the joyful things in life, comparing themselves with others whose health status was worse than their own, standing up for themselves, helping others or maintaining normalcy [10,11,12].

Since many nursing home residents spend the remainder of their life in the nursing home, preserving personal dignity has become an important goal of the care given here [13-16]. To better help nursing home residents maintaining personal dignity throughout their entire admission period, it is valuable to gain insight into the way nursing home residents’ views on dignity develop over the course of time. Chochinov et al. pointed out that as advancing illness fluctuates in its clinical presentation, perceptions of dignity may be similarly dynamic and subject to change [17]. In addition, mechanisms such as perceptual adjustment [18] - methodologically framed as response shift [19] - may play a role in providing nursing home residents with new strategies and regaining control over their new living situation.

Most research on personal dignity has been carried out cross-sectionally [6,11], or, in case of a longitudinal approach, mainly focused on increasing the trustworthiness of a proposed model [10,20]. Until now, it has never been studied if, and in what way, nursing home residents’ personal dignity changes when they reside in the nursing home for a longer period of time. Therefore, the aim of this study is to investigate how personal dignity of nursing home resident develops over the course of time, and what contributes to this.

## Methods

### Ethics Statement

The study was approved by the Medical Ethics Committee of the VU University Medical Center, Amsterdam. Besides, the Management Teams and Clients’ Councils in the nursing homes gave their approval for the research to be carried out. Written - and in a few cases oral if a resident was not able to sign - informed consent to participate in the study and publish the results was obtained of all respondents at each interview appointment.

### Design

A longitudinal qualitative study, in which multiple in-depth interviews with nursing home residents were performed and analysed following the principles of thematic analysis [21].

### Study population

This study is a continuation of a primary study involving 30 nursing home residents from four nursing homes in the Netherlands [12]. Shortly after they were admitted to the nursing home, these residents were interviewed about factors that influenced their personal dignity. In the sampling procedure, we aimed at reaching maximum variation among the characteristics that could potentially influence personal dignity, both in selecting the nursing homes residents (gender, age, religion, cultural background and type of illness) and the different nursing homes (location and privacy conditions) [22,23]. Only residents who were recently admitted to a long-stay unit for residents with physical diseases, able to understand the study and give informed consent, and speak comprehensibly in Dutch were asked to participate in the study. Because of the complex subject matter of the interviews, residents with severe dementia were excluded. To study developments in dignity over the course of time, we also excluded residents on rehabilitation wards, whose length of stay is often short.

From this first interview on, we aimed to follow these residents over the course of time for 18 months, and interviewed them at an interval of six months, until the resident deceased, withdrew from the study due to another reason or until the data collection period ended. In some occasions, in close deliberation with an elderly care physician, nurse or unit manager, this interval was shortened, e.g. when a resident’s health status deteriorated rapidly or when a resident moved to another unit. We were able to interview 22 residents multiple times (two to five times) and conducted 83 interviews with them in total. Reasons for the drop out of the other eight residents were: five residents died in between the first and second interview, one resident had become severely demented, one was transferred to another nursing home and one resident considered the interviews as too burdensome. Among the 22 participating residents, 14 were women and 8 men, ranging in age from 49 to 97 years of age, the mean age being 77 years at the time of the first interview appointment. Table 1 shows the nursing home residents’ characteristics and the number of times they were interviewed.

**Table 1:** Characteristics of respondents.

**Respondent**	**Characteristics**			**Number of interviews**
	**Sex**	**Age range^1^**	**Illness(es**)	
1	Woman	81-90	CVA, COPD, rheumatoid arthritis	4
2	Man	≤ 60	CVA	3*
3	Woman	≤ 60	Crohn’s disease	4
4	Woman	71-80	Not able to stand as a result of trauma	4
5	Woman	61-70	CVA, COPD	4
6	Man	71-80	CVA	4
7	Man	61-70	Multiple system atrophy	4
8	Man	81-90	Heart failure	4
9	Woman	81-90	Arthrosis	4
10	Man	> 90	Rheumatoid arthritis	4
11	Woman	81-90	CVA, heart failure	3^†^
12	Woman	81-90	Huntington’s disease	4
13	Man	71-80	CVA	4
14	Woman	81-90	CVA, diabetes, aneurysm of aorta	5
15	Woman	61-70	Cerebral hemorrhage as a result of trauma	5
16	Woman	> 90	Heart failure	2^†^
17	Man	81-90	Proximal muscle weakness	2^†^
18	Woman	71-80	Myocardial infarction, heart failure, poliomyelitis	4
19	Woman	81-90	CVA	4
20	Woman	81-90	*Arthrosis, osteoporosis, Transient Ischaemic Attacks*	3*
21	Man	71-80	Paralyzed as a result of trauma, diabetes	4
22	Woman	≤ 60	Hydrocefalus as a result of an aneurysm in brains	4

1 Age at the time of the first interview appointment.

† deceased within data collection period.

* withdrew from the study.

CVA: Cerebrovascular accident.

COPD: Chronic obstructive pulmonary disease.

### Data collection

Interviews were conducted from May 2010 to December 2012 and were guided by a topic list. In the first interview, nursing home residents were questioned about their view on personal dignity and the factors that undermined or preserved this. The subsequent interviews mainly consisted of the same questions, but focused on changes in health status, living circumstances or coping capacities, and its relation to perceived dignity. All interviews were performed by the first author, took place in the nursing homes and were recorded and transcribed verbatim. The interviews lasted approximately 45-60 minutes. The interviewer kept field notes, describing her reflections on the interviews and the study.

### Data analysis and rigour

Data analysis started during data collection and was an ongoing process. We coded and analyzed the transcripts with the aid of Atlas-ti software. In the first stage of analysis the interviews were re-read and codes were ascribed to the text sections, following thematic analysis [21]. To ensure reliability of the coding procedure, some interviews were independently coded by the first and third author, which revealed high consensus. In the coding procedure, special attention was paid to text sections in which a resident reported on changes in personal dignity and their cause. To also trace the changes in personal dignity that were not explicitly mentioned by the respondents, all interviews of each resident were read and re-read as a narrative. For each resident, codes were compared per interview and a summary of factors that influenced a resident’s dignity over time was written. These summaries and codes were discussed with the other authors, who are all experienced in performing qualitative research. Overarching themes (e.g. regaining control over one’s life and being regarded as a worthwhile person) were discovered and further questioned in the following interviews, until the research question could be satisfactorily answered. To organize and describe our findings, we made use of the framework of the Model of Dignity in Illness [24]. This model illuminates how illness might affect personal dignity via one or more of three intermediary domains of one’s self – the individual self, relational self and/or the societal self – and that an individual’s coping capacities, a supportive social network and good professional care can protect against threats to personal dignity arising from the different illnesses. This model has been proven to be applicable to the nursing home setting as we earlier used this model’s framework to describe and organize the results of our primary study [12].

## Results

### Changes in personal dignity over time

No general tendency in the development of personal dignity among all participants was found; some residents experienced an improved sense of dignity, while others experienced a downward trend, a fluctuating one or no change at all. To illustrate these different developments, tables 2, 3 and 4 describe the experiences of three nursing home residents. To unravel the factors that contributed to dignity over the course of time, we need to take a closer look at the several developments that nursing home residents experienced during the period of the study, both in the individual, relational and societal domain – and their relation to dignity.

**Table 2:** No change in personal dignity: Mr. 10.

Mr. 10 is over 90 years old when he is admitted to the nursing home, after a period of being in hospital because of a bacterial infection. He has travelled a lot, and therefore feels imprisoned in the nursing home. He has difficulties with living in a small room and sharing the bathroom. Furthermore, he misses liveliness, thinks his view from the window is boring, and wishes to converse more with other people (he has no children, most of his friends died, he cannot get along with other residents, and the nurses have not much time to talk). These factors violate his dignity. In between the 2nd and 3rd interview, Mr. 10 is relocated to another nursing home nearby, where he has his own living room, separate bedroom, kitchenette and bathroom for himself. Whereas the small room first seemed to violate his dignity, more space does not necessarily enhance his sense of dignity now. Although he is pleased with it, a restless feeling remains. This has to do with the structure in the nursing home, of which he feels he is ought to comply with, but at the same time he is tired of adjusting at his age: “*Well, dignity… I really have to adjust again to everything that goes on here. No, I don’t feel that I’m my own person at all anymore. Because now I’m often forced to be somewhere at a certain time. I’m not in control of the appointments. The times here are determined by the dentist, the optician, everything has to be arranged… And all I can do is say whether I can manage to be there or not.*” His restless feeling has also to do with all nurses coming in just like that so that his private life has disappeared, and the long waiting times for help; the same factors that threatened his dignity in the first interview. And these factors continue to threaten his dignity in the 4th interview as well.In short, Mr. 10 expected his dignity to be enhanced in a new living environment (nicer people, a bigger room), but the violation of his dignity appears to be more strongly influenced by his individual difficulties to cope with adjustments: “*It [sense of dignity*]* has not much changed [since moving to another nursing home*]*. It’s just a question of… How should I put it… I’ve spent ten years living independently, alone, and now I’m amongst other people, so my dignity has gone.*”

**Table 3:** A positive change in personal dignity: Mrs. 18.

Mrs. 18 is 71-80 years old, and suffers from heart failure and poliomyelitis. Shortly after her husband died, she got a myocardial infarction, after which she is no longer able to care for herself anymore, and she ends up in a wheelchair. Being tied to a wheelchair undermines her dignity, because she cannot go anywhere she wants without help, which makes her feel a burden to the nurses. Looking well-groomed is important for her dignity, as well as having contacts with others. However, making new contacts in the nursing home appears disappointing at first, as most other residents are cognitively impaired or cannot talk at all. After 6 months, Mrs. 18 has become more content with her life in the nursing home. She has furnished her room with her own stuff, enjoys all activities that are organized and meets with other nursing home residents whom she likes. These social aspects have a positive bearing on her sense of dignity. Mrs. 18 reflects: “*I was terribly homesick the first fortnight. I just wanted to go home. I found all the people equally decrepit. Okay, I’m not well because I can’t walk and all that, but I’m still mentally all right. And I thought oh how terrible, do I have to spend my last years of life here like this? But after a couple of weeks, they had soon brought me my own things and that… Funny isn’t it? That was really a part of me, and that cheered me up a bit and I hung up some curtains. But after I got my own things back, well that made it a bit better, then it was okay. But now I am really enjoying life here. Yes because the good thing about this place is that we have such a lot of activities. And that is sociable because then we are all together, all the people from all the different parts of the complex. And there are such a lot of people at the moment who I enjoy chatting to*.” Another positive effect on residing longer in the nursing home is that is becomes easier for Mrs. 18 to have to rely on the nursing home staff and to ask them something. The nurses can help her to maintain her dignity when she loses urine, by responding as if it is no problem at all: “*In the beginning, oh I found it so terrible, my underwear would be a bit wet and they would say: don’t worry about it, that happens all the time here, I’ll get you a new pair. It’s just as easy. Personally, I found it disagreeable every time. I think oh no, I’ve done it in my underpants again. But well… They don’t mind. And they will never say do you need to go to the bathroom yet again, or do you need to do this or that yet again…*” Mrs. 18’s health status remains stable during the study period. However, in between the 2nd and 3rd interview, Mrs. 18 receives an electric wheelchair. This improves her dignity, because she is less dependent now: “*I function better now. Then I was sitting in a wheelchair and they had to push me everywhere. Then I had no choice but to sit here, so there I was waiting… They would come and get me when it was time to eat, and now I can get around everywhere myself. So no, it has changed. I now definitely feel I have more dignity than then.*” In addition, by saying good-bye to her former house gradually, Mrs. 18 was able to adjust and accept her new life in the nursing home more and more. This helped her in regaining her dignity. She even says: “*Yes, the nursing home isn’t so bad. Well, I have been relieved of a huge burden, otherwise I would have been left alone; my husband had died shortly before. And then you are there alone in a big apartment, you’re sitting there alone and you just have to wait until the kids come along, as it were.*”

**Table 4:** A declining personal dignity: Mrs. 20.

Mrs. 20, a woman of 81-90 years old with arthrosis and osteoporosis, is unhappy and feels undignified in the nursing home. She feels as if she is snatched away from her normal life, and she does sometimes not recognize herself anymore, because of her changed voice due to TIAs. Her dignity is consequently violated, and also by the way some nurses treat her: unfriendly and commanding. In addition, throughout the study period, she experiences a negative atmosphere on the ward. She feels that the nurses and other residents regard her as a grumbler, and she does not know how to get rid of this image. This causes her to behave more introvert as she used to be: “*Yes, I can’t really be myself. I’m the type who always likes a giggle, and you should see what it’s like here… No, that’s more the consequence of my condition; I can’t be like that anymore. Then I’m sitting there groaning a bit, that spot hurts so much, you know… Then* I suspect *they think of you as a bit of a fusspot. So I prefer just to be sitting somewhere quietly in the café. I don’t think you should always be doing that, but otherwise you just get more squabbling - no thank you*.” During the study period, Mrs. 20 health status decreases. Whereas she walks behind a stroller in the first interview, she needs a wheelchair 6 months later. This negatively influences her dignity: “*Now I’m completely at their mercy. I sit here and I just have to sit here and watch what happens. So now I feel I’m completely worthless. But I can’t mess around, can’t do anything, I can’t go anywhere. No, you have to ask for everything, you have to wait and see for everything. You are completely worthless.*” Also becoming more forgetful makes her feel less dignified, because it points out her deterioration. Visitors however can cheer Mrs. 20 up. She can then be herself completely, because these people know how she was before her illness. However, as these other people also grow older, become less mobile or have busy lives, after a year they do not come as often as in the beginning. She therefore experiences this positive influence on dignity less frequently: “*I feel worthless, except when I have a visitor from outside, from the old days, the occasional person… Of course there’s not so many of them left, they are dropping off too. But if they are there, old friends or neighbors who come along to see me, then I’m in seventh heaven as it were, those are my people. Then I feel I have my dignity, those people who come for me. And I think that’s wonderful. I always reckon that they at least know who I am.*”

### Illness related factors

Health status could deteriorate or improve over the course of time. In our sample, small deteriorations in health occurred, but there were not many people whose health deteriorated heavily. Only when more loss of autonomy was experienced, for instance by becoming bound to a wheelchair (as happened for Mrs. 20 - see table 4) or to bed, residents reported a declined personal dignity and sometimes even indicated that their lives were not worth living anymore. In some occasions, due to regular physiotherapy, a small improvement in health was experienced, e.g. more strength in arms or legs. This could restore a resident’s dignity, because it gave them prospects, hope and something to fight for, e.g. for more autonomy and freedom.

*Respondent 5: Well, it [sense of dignity*]* was worse then [six months ago*]*. Yes, much worse. Yes, well not any more - you get your feeling of dignity back after a bit, don’t you? Because now I go home on my own in the minibus. And then I go upstairs on my own, along the access balcony, I go back down, get the minibus at seven o'clock, it’s all those little things that I’m able to do again. Yes, I’ve been able to get a little bit of grip back on my life again.*

### The individual self

With regard to the individual’s internal evaluation and one’s perception of having worth as an individual, nursing home residents reported that getting used to the structures and the way things were handled in the nursing home over the course of time could help them to become more self-assured and feel at ease. This could positively influence their dignity.

*Respondent 22: Then [when I had just arrived in the nursing home*]* I felt I had lost some of my dignity. I had to get used to it. So that makes you feel unsure of yourself - I didn’t know how things worked, so I felt inferior. But I soon got my feeling of dignity back. Then you realize you aren’t inferior to the rest and then it comes back.*

In addition, time generally contributed to the extent to which residents could accept their situation. Whereas several residents struggled with their situation in the first interview, they were often much milder about their physical condition and admission later on. Nursing home residents reported that they got used over time to receive help with washing and dressing, which lessened their feelings of embarrassment. They frequently came to terms with their situation, by stating that living at home was no longer possible and that they would have been lonely there (see table 3). As such, this could restore their sense of dignity.

*Interviewer: But, talking about personal dignity, is that different now to how it was a year ago, when you had just arrived here [in the nursing home*]*?*

*Respondent 19: Yes, very different... It’s because you are more likely to resign yourself to those things, because there’s no alternative. I should be pleased I’m here at all. And I am. Because I couldn't have gone home again in the early days... I can’t even go to the bathroom on my own; I tried it once and luckily [name of male nurse*]* caught me in time otherwise I would’ve been lying there next to the toilet bowl. And I’m pleased I can still stand, I’m very pleased about that.*

Another coping mechanism that we could discern from the interviews was that several residents became better able to place their own situation in perspective. Statements like “So it could have been much worse” (respondent 12) and “I’m proud I’ve still got all my marbles” (respondent 6) were more present in the subsequent interviews than in the first one. Also by comparing themselves with others who malfunctioned worse, residents regained a focus on things in life they till could do, instead of all things they could not do anymore. This helped them to regain a feeling of control over their lives, and could preserve their dignity.

Medical treatment could in some cases enhance a resident’s autonomy. Several residents received for example an electric wheelchair during the study period, which they all evaluated as an enhancement of autonomy, and could positively influence their personal dignity (see table 3). It heightened their freedom of movement and made them less dependent on others, so that they had not to ask the nurses for all sort of things.

We noticed in the interviews that personal dignity was not much influenced by the features of the room the resident lived in (e.g. amount of space, modern appearance). For instance, respondent 15, who first shared a room with others and was later relocated to a new location with her own spacious room, said that having her own room was pleasant, but not enhancing her personal dignity. Feeling a burden to her family and not being able to help her daughter with raising her children remained the same in both locations. Also Mr. 10’s dignity did not benefit from a relocation to another room (see table 2). As such, the nursing home can facilitate certain aspects (e.g. stimulating autonomy), but a person’s character and values also play an important role.

### The relational self

In the realm of the relational self, analysis of the interviews revealed that residing longer in a nursing home could contribute to the number of new acquired contacts. New contacts with other residents were often made during organized activities, and added to a sense of belonging, gave life more meaning, and could enhance personal dignity (see table 3). In addition, getting familiarized with the nursing home staff could help residents to feel more convenient to display their wishes:

*Respondent 22: They treat you with respect. They do now, but not at the start because you hadn’t been around so long. If there’s something I want now, they will consider it or discuss it. Before, they would've said no straight away. They don’t care two hoots about you. That has got better over time, them respecting your wishes. For example, how I want to be washed, the fact that I don’t always want to take a shower, that I don’t always want to eat in the dining room.*

*Interviewer: And did they just take you to the dining room before, without asking you whether you actually wanted to go? Or did you say what you wanted before?*

*Respondent: No, I didn’t do that either. Maybe it’s something that has to come from both sides.*

We also found that a lot of nursing home residents became milder about the nurses over the course of time. Whereas waiting for help was an important aspect undermining dignity in the first interview – because residents felt neglected or could not make it to the toilet in time – it became less important later on as residents gained more understanding that they were not the only one who needed help. Nevertheless, waiting for help remained a frequently mentioned aspect when residents were asked what could improve in the nursing home as to enhance their dignity.

The way in which nurses responded to potentially embarrassing situations, e.g. accidentally losing urine, was important for the preservation of dignity. When nursing home residents discovered that nurses reacted very naturally as if losing urine was perfectly normal, their feelings of being a burden decreased and it helped to regain their dignity (see table 3).

*Respondent 4: Yes, I’ve reached that stage now. Of course that’s something you have to accept, that kind of thing [accidently losing urine*]*. Because if you’re not used to it and suddenly all that’s happening to you, then you feel… After all, you’re burdening someone else with all your mess.*

*Interviewer: So is it more degrading the first time something like that happens than when it happens more often?*

*Respondent: Yes, because you see how they deal with it and that they find… Of course you get an awful lot of people coming along, there are so many people helping you, different people. And yes, they’re all very relaxed about it, so that obviously makes a huge difference. So you think, well, it has to be done, and they are so used to it, so they can’t find it that awful.*

Lastly, some residents mentioned that the longer they resided in the nursing home, the more they got forgotten by family and friends. Whereas they received many visitors in the first few weeks, they got less frequent visits later on, because potential visitors were too busy, also became more impaired or died (see table 4). This notion could make residents feel worthless and undermined their dignity.

*Respondent 9: There is nothing left of your dignity… No. No, the people… No, well he’s just lying in the nursing home… Just let him lie there… No, it’s not much fun anymore, you know. Or gosh, I have to go there as well, you know. And then immediately put it to one side and forget about it. You are alive but you aren’t really living any more.*

### The societal self

Aspects in the domain of the societal self were less frequently mentioned as threatening dignity over the course of time. Whereas several nursing home residents revealed that they felt redundant, stigmatized and an economical burden to society in the first interview, most of them did not bring this up in the subsequent interviews. This is probably because living in a nursing home protected them against exposures to disrespect and societal discourses on ageism in the outer world. In addition, being amongst disabled others caused residents to feel less deviant from others in what has become their ‘new’ small society.

*Respondent 21: Yes, you have to fight for it. Especially people like me… Really have to fight for a position in society… So yes, you have to create your own dignity, show them that you’re really not backward or anything. They don’t do that here [in the nursing home*]*. But they do in society, you know, they are quick to look down on you. They are very quick to do that.*

## Discussion

We found that two mechanisms were especially important for a nursing home resident to maintain or regain personal dignity: the feeling that one is in control of his life and the feeling that one is regarded as a worthwhile person, both by themselves and by others. The acquirement of both feelings can be supported by 1) finding a way to cope with one’s situation, 2) getting acquainted with the new living structures in the nursing home and therefore feeling more at ease, 3) physical improvement (with or without an electric wheelchair), 4) being socially involved with nursing home staff, other residents and relatives and 5) being amongst disabled others and therefore less prone to exposures of disrespect from the outer world.

The feeling of being in control of one’s life appears not only important for restoring personal dignity, but has elsewhere been mentioned as contributing to become satisfied with living in a long-term care facility over time [7]. To realise this, our study population used both emotion-focused and, to a lesser extent, problem-focused coping strategies [8,9]. Emotion-focused coping occurred on the part of the individual resident (e.g. by reassessing the situation and adjusting to it), while problem-focused coping was more often initiated by the nursing home staff (e.g. providing the resident with physiotherapy or an electric wheelchair).

Brooke identified and described four adaptation phases as required for a successful transition into a long-term care facility - disorganization, reorganization, relationship building and stabilization [25]. We observed in our study that the restoration of personal dignity could go along with these phases, as can be perceived in Mrs. 18’s story (table 3). After an initial short phase of complete desperation (disorganization), this resident’s own furniture helped her to settle down (reorganization); and getting familiar with the nursing home staff and other residents (relationship building) contributed to her finding a new, satisfying perspective on her life in the nursing home (stabilization). Comparably, many nursing home residents seemed to gradually perceptually adjust to their new situation and came to accept potential indignities (e.g. losing urine or waiting for help) without the loss of dignity that they might have anticipated when thinking about these matters earlier in life [18].

Nevertheless, these success stories do not apply to all residents. We observed that the time spent in one particular adaptation phase varied widely between the nursing home residents, and a few residents did not shift between phases at all. For example, we noticed that nursing home residents who strongly emphasized being independent as a requirement to feel dignified had more difficulties with accepting their situation and regaining dignity over the course of time. For these nursing home residents, personal dignity did barely change, even though the living circumstances in the nursing home sometimes had changed.

### Strengths and limitations of the study

An important strength of this study is that it is the first one, to our knowledge, investigating nursing home residents’ views on dignity over a longer period of time. Besides generating a longitudinal perspective, conducting repeated interviews had two advantages: we could discuss certain aspects again if they were insufficiently discussed in a former interview, resulting in greater reliability, and it could create more rapport between the interviewer and respondent, which might lead to less social-desirable answers and therefore more validity. The latter advantage would however have been greater in other populations, because a lot of nursing home residents had great difficulties to remember what the earlier interviews were about. A limitation of the study is that we only investigated the developments in personal dignity for residents on general medical wards. Although some residents in our study suffered from the early stages of dementia, we do not know whether our results can be generalized to residents with more severe dementia. Furthermore, we were not able to thoroughly study the influence of a changed health status on personal dignity, because only minor deteriorations or improvements occurred in this severely impaired nursing home population. Whether the perceptions of dignity are dynamic as advancing illness fluctuates in its clinical presentation [17], or, contrary, do not substantially change as health status changes [26] needs further investigation in other populations.

### Conclusions and recommendations for practice

This study shows that personal dignity can change over the course of time. It does not just diminish by residing longer in a nursing home. Instead, several residents managed to perceptually adjust to their new situation, causing them to experience their admission as bearable and helping them to regain their dignity over the course of time. Some eventually considered to have more dignity while living in the nursing home than they would have had if they had stayed home. The feeling that one is in control of his life and the feeling that one is regarded as a worthwhile person seem to be key mechanisms in this.

Besides above-mentioned positive consequences, the ability to perceptually adjust brings on the risk that potentially degrading situations may become too easily accepted by residents as well as nursing home staff. Therefore, we would like to emphasize that these findings do by no means imply that nursing home staff can just wait for a resident to adjust and regain dignity; good professional care requires staff to take an active part in helping residents with this process. This study has given insight into ways nursing home staff can facilitate and create optimal conditions to help a nursing home resident recover above mentioned feelings; by intervening in the different domains described in the Model of Dignity in Illness [24]. For example, a psychologist could help a nursing home resident to reinforce coping skills in order to obtain a new perspective on life. Additionally, it seems important to explain the structures, habits and activities that the nursing home offers soon after admission. This might accelerate the process of settling down and, hence, enhances feelings of mastering the situation. Furthermore, nursing home staff should support residents to have as much autonomy as possible, e.g. by providing an electric wheelchair or listen seriously to a resident’s wishes. Finally, families should be encouraged to visit the nursing home resident not only after recent admission, but also after a longer period of time. Nevertheless, despite the efforts of nursing home staff, whether or not a resident succeeds to retrieve personal dignity appears also to be dependent on a resident’s character, and always takes time.
